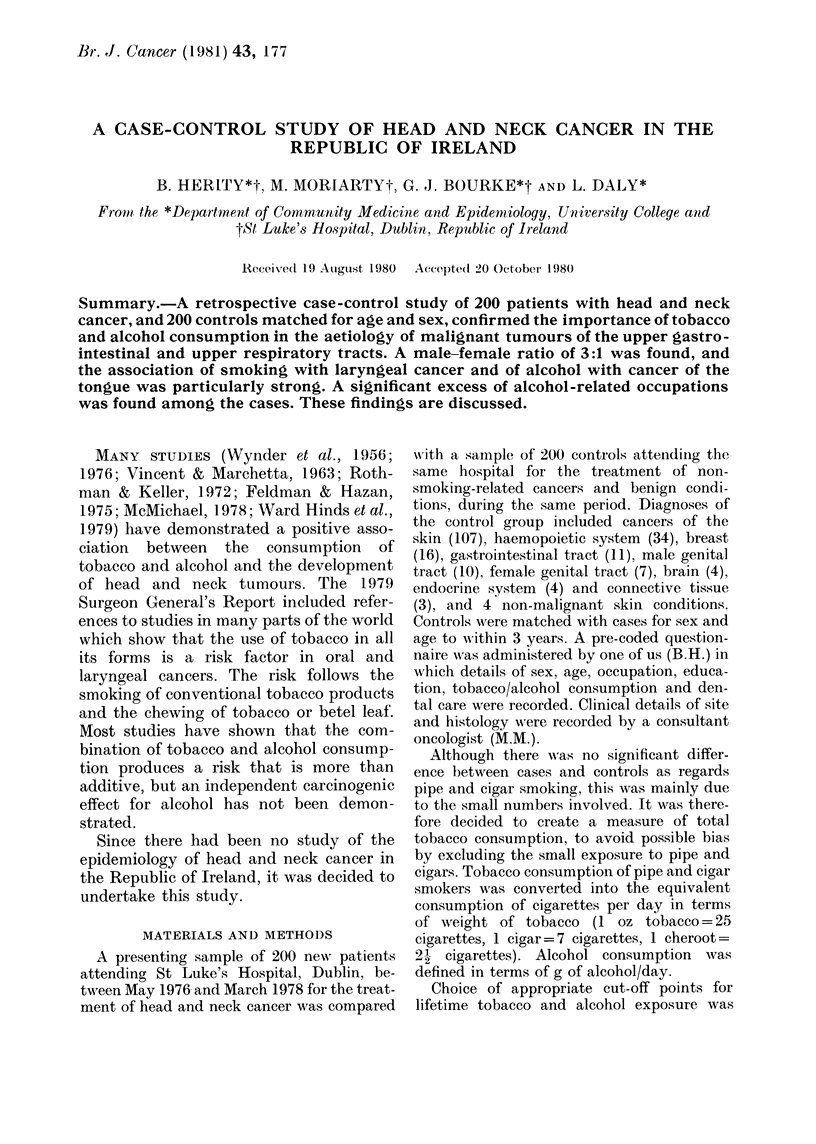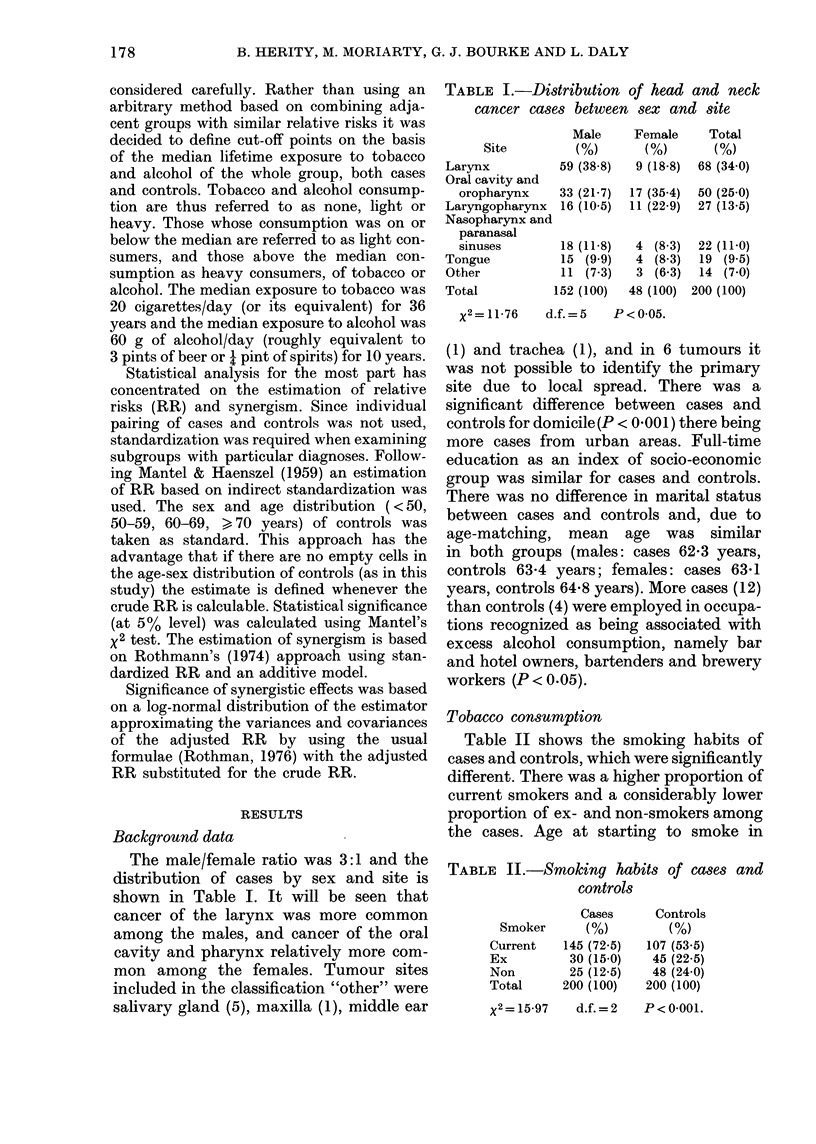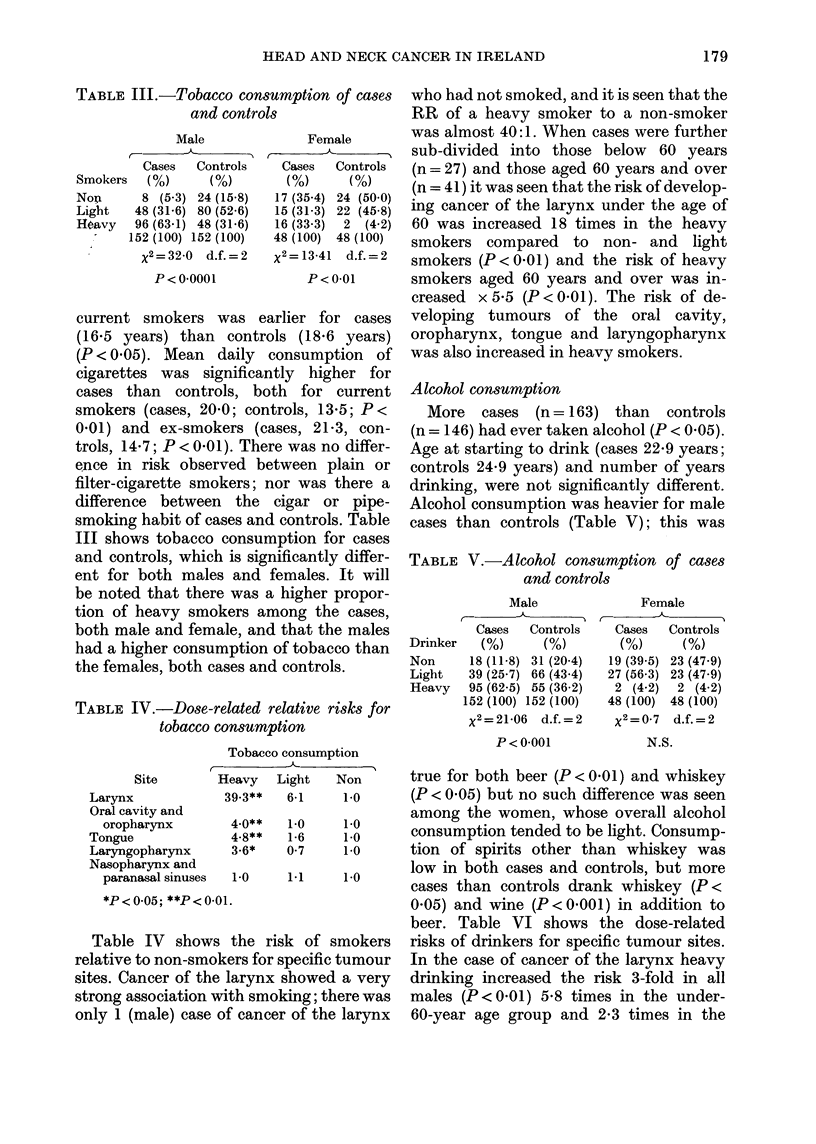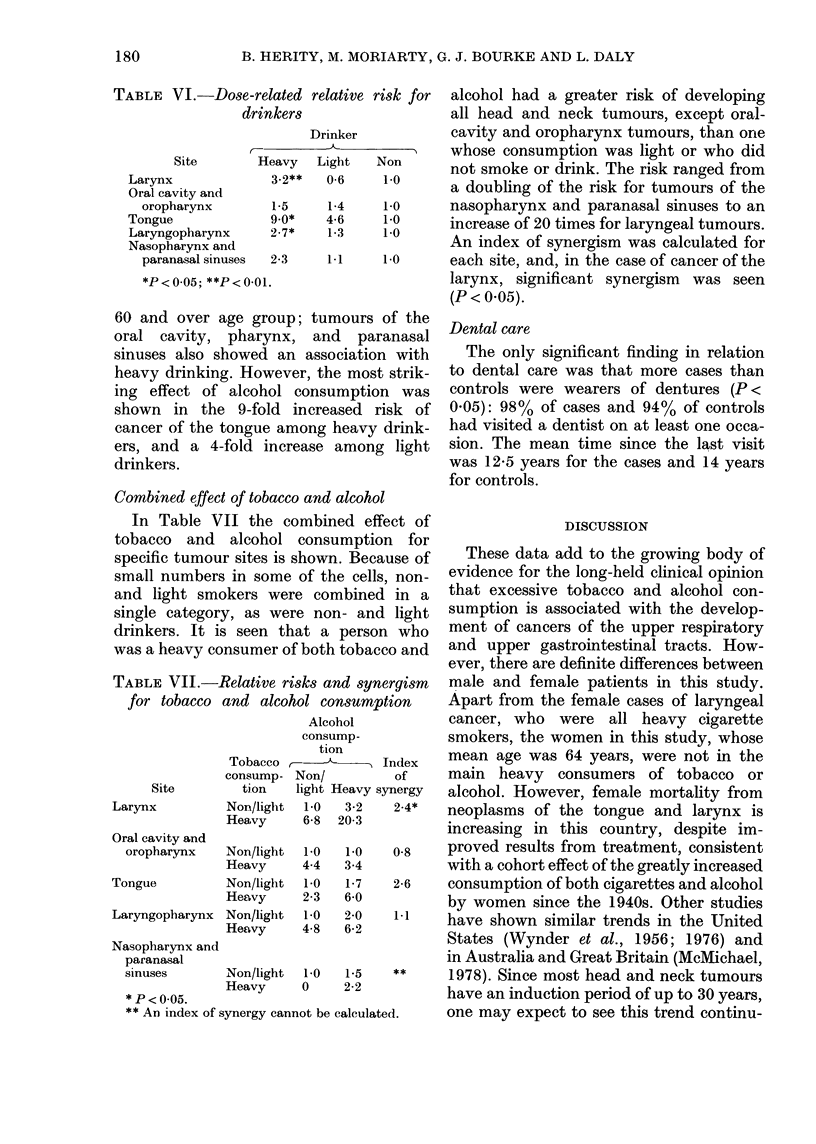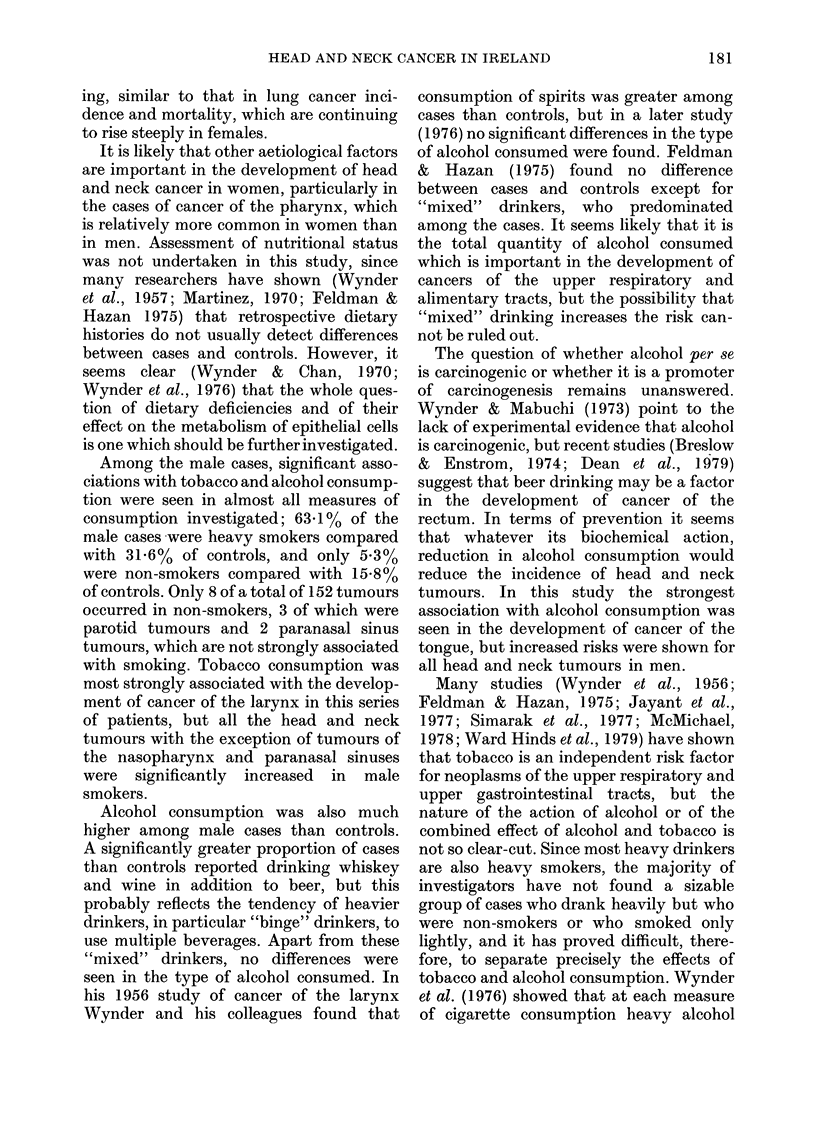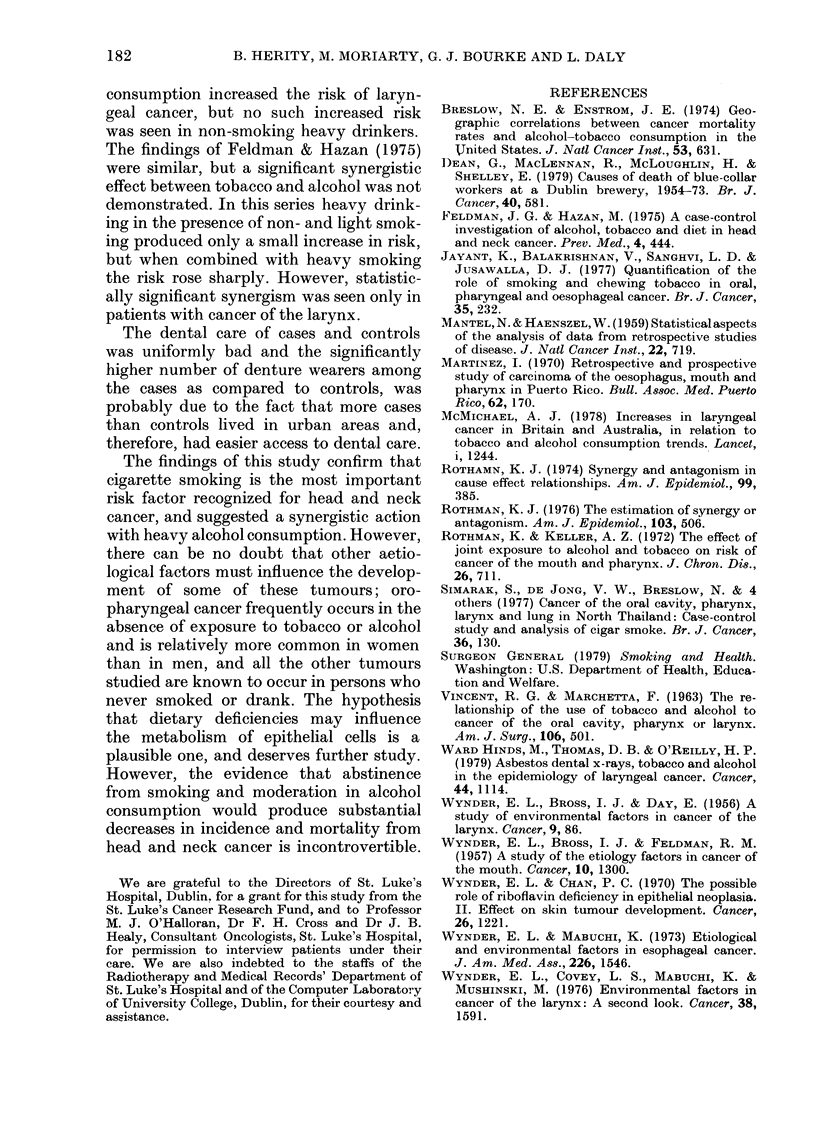# A case-control study of head and neck cancer in the Republic of Ireland.

**DOI:** 10.1038/bjc.1981.26

**Published:** 1981-02

**Authors:** B. Herity, M. Moriarty, G. J. Bourke, L. Daly

## Abstract

A retrospective case-control study of 200 patients with head and neck cancer, and 200 controls matched for age and sex, confirmed the importance of tobacco and alcohol consumption in the aetiology of malignant tumours of the upper gastrointestinal and upper respiratory tracts. A male-female ratio of 3:1 was found, and the association of smoking with laryngeal cancer and of alcohol with cancer of the tongue was particularly strong. A significant excess of alcohol-related occupations was found among the cases. These findings are discussed.


*Br. J. Cancer* (1981) **43**, 177

### A CASE-CONTROL STUDY OF HEAD AND NECK CANCER IN THE REPUBLIC OF IRELAND

B. HERITY*†, M. MORIARTY†, G. J. BOURKE*† AND L. DALY*

*From the *Department of Community Medicine and Epidemiology, University College and †St Luke's Hospital, Dublin, Republic of Ireland*

Received 19 August 1980 Accepted 20 October 1980

**Summary.—A retrospective case-control study of 200 patients with head and neck cancer, and 200 controls matched for age and sex, confirmed the importance of tobacco and alcohol consumption in the aetiology of malignant tumours of the upper gastro-intestinal and upper respiratory tracts. A male–female ratio of 3:1 was found, and the association of smoking with laryngeal cancer and of alcohol with cancer of the tongue was particularly strong. A significant excess of alcohol-related occupations was found among the cases. These findings are discussed.**

MANY STUDIES (Wynder *et al.*, 1956; 1976; Vincent & Marchetta, 1963; Rothman & Keller, 1972; Feldman & Hazan, 1975; McMichael, 1978; Ward Hinds *et al.*, 1979) have demonstrated a positive association between the consumption of tobacco and alcohol and the development of head and neck tumours. The 1979 Surgeon General's Report included references to studies in many parts of the world which show that the use of tobacco in all its forms is a risk factor in oral and laryngeal cancers. The risk follows the smoking of conventional tobacco products and the chewing of tobacco or betel leaf. Most studies have shown that the combination of tobacco and alcohol consumption produces a risk that is more than additive, but an independent carcinogenic effect for alcohol has not been demonstrated.

Since there had been no study of the epidemiology of head and neck cancer in the Republic of Ireland, it was decided to undertake this study.

### MATERIALS AND METHODS

A presenting sample of 200 new patients attending St Luke's Hospital, Dublin, between May 1976 and March 1978 for the treatment of head and neck cancer was compared with a sample of 200 controls attending the same hospital for the treatment of non-smoking-related cancers and benign conditions, during the same period. Diagnoses of the control group included cancers of the skin (107), haemopoietic system (34), breast (16), gastrointestinal tract (11), male genital tract (10), female genital tract (7), brain (4), endocrine system (4) and connective tissue (3), and 4 non-malignant skin conditions. Controls were matched with cases for sex and age to within 3 years. A pre-coded questionnaire was administered by one of us (B.H.) in which details of sex, age, occupation, education, tobacco/alcohol consumption and dental care were recorded. Clinical details of site and histology were recorded by a consultant oncologist (M.M.).

Although there was no significant difference between cases and controls as regards pipe and cigar smoking, this was mainly due to the small numbers involved. It was therefore decided to create a measure of total tobacco consumption, to avoid possible bias by excluding the small exposure to pipe and cigars. Tobacco consumption of pipe and cigar smokers was converted into the equivalent consumption of cigarettes per day in terms of weight of tobacco (1 oz tobacco = 25 cigarettes, 1 cigar = 7 cigarettes, 1 cheroot = $2\frac{1}{2}$ cigarettes). Alcohol consumption was defined in terms of g of alcohol/day.

Choice of appropriate cut-off points for lifetime tobacco and alcohol exposure was considered carefully. Rather than using an arbitrary method based on combining adjacent groups with similar relative risks it was decided to define cut-off points on the basis of the median lifetime exposure to tobacco and alcohol of the whole group, both cases and controls. Tobacco and alcohol consumption are thus referred to as none, light or heavy. Those whose consumption was on or below the median are referred to as light consumers, and those above the median consumption as heavy consumers, of tobacco or alcohol. The median exposure to tobacco was 20 cigarettes/day (or its equivalent) for 36 years and the median exposure to alcohol was 60 g of alcohol/day (roughly equivalent to 3 pints of beer or $\frac{1}{4}$ pint of spirits) for 10 years.

Statistical analysis for the most part has concentrated on the estimation of relative risks (RR) and synergism. Since individual pairing of cases and controls was not used, standardization was required when examining subgroups with particular diagnoses. Following Mantel & Haenszel (1959) an estimation of RR based on indirect standardization was used. The sex and age distribution ($<50$, 50–59, 60–69, $\geqslant 70$ years) of controls was taken as standard. This approach has the advantage that if there are no empty cells in the age-sex distribution of controls (as in this study) the estimate is defined whenever the crude RR is calculable. Statistical significance (at 5% level) was calculated using Mantel's $\chi^2$ test. The estimation of synergism is based on Rothmann's (1974) approach using standardized RR and an additive model.

Significance of synergistic effects was based on a log-normal distribution of the estimator approximating the variances and covariances of the adjusted RR by using the usual formulae (Rothman, 1976) with the adjusted RR substituted for the crude RR.

### RESULTS

*Background data*

The male/female ratio was 3:1 and the distribution of cases by sex and site is shown in Table I. It will be seen that cancer of the larynx was more common among the males, and cancer of the oral cavity and pharynx relatively more common among the females. Tumour sites included in the classification "other" were salivary gland (5), maxilla (1), middle ear (1) and trachea (1), and in 6 tumours it was not possible to identify the primary site due to local spread. There was a significant difference between cases and controls for domicile ($P < 0{\cdot}001$) there being more cases from urban areas. Full-time education as an index of socio-economic group was similar for cases and controls. There was no difference in marital status between cases and controls and, due to age-matching, mean age was similar in both groups (males: cases 62·3 years, controls 63·4 years; females: cases 63·1 years, controls 64·8 years). More cases (12) than controls (4) were employed in occupations recognized as being associated with excess alcohol consumption, namely bar and hotel owners, bartenders and brewery workers ($P < 0{\cdot}05$).

TABLE I.—*Distribution of head and neck cancer cases between sex and site*

| Site | Male (%) | Female (%) | Total (%) |
|---|---|---|---|
| Larynx | 59 (38·8) | 9 (18·8) | 68 (34·0) |
| Oral cavity and oropharynx | 33 (21·7) | 17 (35·4) | 50 (25·0) |
| Laryngopharynx | 16 (10·5) | 11 (22·9) | 27 (13·5) |
| Nasopharynx and paranasal sinuses | 18 (11·8) | 4 (8·3) | 22 (11·0) |
| Tongue | 15 (9·9) | 4 (8·3) | 19 (9·5) |
| Other | 11 (7·3) | 3 (6·3) | 14 (7·0) |
| Total | 152 (100) | 48 (100) | 200 (100) |

$\chi^2 = 11{\cdot}76$ d.f. $= 5$ $P < 0{\cdot}05$.

*Tobacco consumption*

Table II shows the smoking habits of cases and controls, which were significantly different. There was a higher proportion of current smokers and a considerably lower proportion of ex- and non-smokers among the cases. Age at starting to smoke in

TABLE II.—*Smoking habits of cases and controls*

| Smoker | Cases (%) | Controls (%) |
|---|---|---|
| Current | 145 (72·5) | 107 (53·5) |
| Ex | 30 (15·0) | 45 (22·5) |
| Non | 25 (12·5) | 48 (24·0) |
| Total | 200 (100) | 200 (100) |

$\chi^2 = 15{\cdot}97$ d.f. $= 2$ $P < 0{\cdot}001$.

TABLE III.—*Tobacco consumption of cases and controls*

| | Male | | Female | |
|---|---|---|---|---|
| Smokers | Cases (%) | Controls (%) | Cases (%) | Controls (%) |
| Non | 8 (5·3) | 24 (15·8) | 17 (35·4) | 24 (50·0) |
| Light | 48 (31·6) | 80 (52·6) | 15 (31·3) | 22 (45·8) |
| Heavy | 96 (63·1) | 48 (31·6) | 16 (33·3) | 2 (4·2) |
| | 152 (100) | 152 (100) | 48 (100) | 48 (100) |
| | $\chi^2 = 32·0$ | d.f. = 2 | $\chi^2 = 13·41$ | d.f. = 2 |
| | $P < 0·0001$ | | $P < 0·01$ | |

current smokers was earlier for cases (16·5 years) than controls (18·6 years) ($P < 0·05$). Mean daily consumption of cigarettes was significantly higher for cases than controls, both for current smokers (cases, 20·0; controls, 13·5; $P < 0·01$) and ex-smokers (cases, 21·3, controls, 14·7; $P < 0·01$). There was no difference in risk observed between plain or filter-cigarette smokers; nor was there a difference between the cigar or pipe-smoking habit of cases and controls. Table III shows tobacco consumption for cases and controls, which is significantly different for both males and females. It will be noted that there was a higher proportion of heavy smokers among the cases, both male and female, and that the males had a higher consumption of tobacco than the females, both cases and controls.

TABLE IV.—*Dose-related relative risks for tobacco consumption*

| | Tobacco consumption | | |
|---|---|---|---|
| Site | Heavy | Light | Non |
| Larynx | 39·3** | 6·1 | 1·0 |
| Oral cavity and oropharynx | 4·0** | 1·0 | 1·0 |
| Tongue | 4·8** | 1·6 | 1·0 |
| Laryngopharynx | 3·6* | 0·7 | 1·0 |
| Nasopharynx and paranasal sinuses | 1·0 | 1·1 | 1·0 |

*$P < 0·05$; **$P < 0·01$.

Table IV shows the risk of smokers relative to non-smokers for specific tumour sites. Cancer of the larynx showed a very strong association with smoking; there was only 1 (male) case of cancer of the larynx who had not smoked, and it is seen that the RR of a heavy smoker to a non-smoker was almost 40:1. When cases were further sub-divided into those below 60 years (n = 27) and those aged 60 years and over (n = 41) it was seen that the risk of developing cancer of the larynx under the age of 60 was increased 18 times in the heavy smokers compared to non- and light smokers ($P < 0·01$) and the risk of heavy smokers aged 60 years and over was increased ×5·5 ($P < 0·01$). The risk of developing tumours of the oral cavity, oropharynx, tongue and laryngopharynx was also increased in heavy smokers.

*Alcohol consumption*

More cases (n = 163) than controls (n = 146) had ever taken alcohol ($P < 0·05$). Age at starting to drink (cases 22·9 years; controls 24·9 years) and number of years drinking, were not significantly different. Alcohol consumption was heavier for male cases than controls (Table V); this was true for both beer ($P < 0·01$) and whiskey ($P < 0·05$) but no such difference was seen among the women, whose overall alcohol consumption tended to be light. Consumption of spirits other than whiskey was low in both cases and controls, but more cases than controls drank whiskey ($P < 0·05$) and wine ($P < 0·001$) in addition to beer. Table VI shows the dose-related risks of drinkers for specific tumour sites. In the case of cancer of the larynx heavy drinking increased the risk 3-fold in all males ($P < 0·01$) 5·8 times in the under-60-year age group and 2·3 times in the

TABLE V.—*Alcohol consumption of cases and controls*

| | Male | | Female | |
|---|---|---|---|---|
| Drinker | Cases (%) | Controls (%) | Cases (%) | Controls (%) |
| Non | 18 (11·8) | 31 (20·4) | 19 (39·5) | 23 (47·9) |
| Light | 39 (25·7) | 66 (43·4) | 27 (56·3) | 23 (47·9) |
| Heavy | 95 (62·5) | 55 (36·2) | 2 (4·2) | 2 (4·2) |
| | 152 (100) | 152 (100) | 48 (100) | 48 (100) |
| | $\chi^2 = 21·06$ | d.f. = 2 | $\chi^2 = 0·7$ | d.f. = 2 |
| | $P < 0·001$ | | N.S. | |

TABLE VI.—*Dose-related relative risk for drinkers*

| Site | Drinker: Heavy | Light | Non |
|---|---|---|---|
| Larynx | 3·2** | 0·6 | 1·0 |
| Oral cavity and oropharynx | 1·5 | 1·4 | 1·0 |
| Tongue | 9·0* | 4·6 | 1·0 |
| Laryngopharynx | 2·7* | 1·3 | 1·0 |
| Nasopharynx and paranasal sinuses | 2·3 | 1·1 | 1·0 |

*$P < 0·05$; **$P < 0·01$.

60 and over age group; tumours of the oral cavity, pharynx, and paranasal sinuses also showed an association with heavy drinking. However, the most striking effect of alcohol consumption was shown in the 9-fold increased risk of cancer of the tongue among heavy drinkers, and a 4-fold increase among light drinkers.

*Combined effect of tobacco and alcohol*

In Table VII the combined effect of tobacco and alcohol consumption for specific tumour sites is shown. Because of small numbers in some of the cells, non- and light smokers were combined in a single category, as were non- and light drinkers. It is seen that a person who was a heavy consumer of both tobacco and alcohol had a greater risk of developing all head and neck tumours, except oral-cavity and oropharynx tumours, than one whose consumption was light or who did not smoke or drink. The risk ranged from a doubling of the risk for tumours of the nasopharynx and paranasal sinuses to an increase of 20 times for laryngeal tumours. An index of synergism was calculated for each site, and, in the case of cancer of the larynx, significant synergism was seen ($P < 0·05$).

TABLE VII.—*Relative risks and synergism for tobacco and alcohol consumption*

| Site | Tobacco consumption | Alcohol consumption: Non/light | Heavy | Index of synergy |
|---|---|---|---|---|
| Larynx | Non/light | 1·0 | 3·2 | 2·4* |
| | Heavy | 6·8 | 20·3 | |
| Oral cavity and oropharynx | Non/light | 1·0 | 1·0 | 0·8 |
| | Heavy | 4·4 | 3·4 | |
| Tongue | Non/light | 1·0 | 1·7 | 2·6 |
| | Heavy | 2·3 | 6·0 | |
| Laryngopharynx | Non/light | 1·0 | 2·0 | 1·1 |
| | Heavy | 4·8 | 6·2 | |
| Nasopharynx and paranasal sinuses | Non/light | 1·0 | 1·5 | ** |
| | Heavy | 0 | 2·2 | |

* $P < 0·05$.
** An index of synergy cannot be calculated.

*Dental care*

The only significant finding in relation to dental care was that more cases than controls were wearers of dentures ($P < 0·05$): 98% of cases and 94% of controls had visited a dentist on at least one occasion. The mean time since the last visit was 12·5 years for the cases and 14 years for controls.

### DISCUSSION

These data add to the growing body of evidence for the long-held clinical opinion that excessive tobacco and alcohol consumption is associated with the development of cancers of the upper respiratory and upper gastrointestinal tracts. However, there are definite differences between male and female patients in this study. Apart from the female cases of laryngeal cancer, who were all heavy cigarette smokers, the women in this study, whose mean age was 64 years, were not in the main heavy consumers of tobacco or alcohol. However, female mortality from neoplasms of the tongue and larynx is increasing in this country, despite improved results from treatment, consistent with a cohort effect of the greatly increased consumption of both cigarettes and alcohol by women since the 1940s. Other studies have shown similar trends in the United States (Wynder *et al.*, 1956; 1976) and in Australia and Great Britain (McMichael, 1978). Since most head and neck tumours have an induction period of up to 30 years, one may expect to see this trend continuing, similar to that in lung cancer incidence and mortality, which are continuing to rise steeply in females.

It is likely that other aetiological factors are important in the development of head and neck cancer in women, particularly in the cases of cancer of the pharynx, which is relatively more common in women than in men. Assessment of nutritional status was not undertaken in this study, since many researchers have shown (Wynder *et al.*, 1957; Martinez, 1970; Feldman & Hazan 1975) that retrospective dietary histories do not usually detect differences between cases and controls. However, it seems clear (Wynder & Chan, 1970; Wynder *et al.*, 1976) that the whole question of dietary deficiencies and of their effect on the metabolism of epithelial cells is one which should be further investigated.

Among the male cases, significant associations with tobacco and alcohol consumption were seen in almost all measures of consumption investigated; 63·1% of the male cases were heavy smokers compared with 31·6% of controls, and only 5·3% were non-smokers compared with 15·8% of controls. Only 8 of a total of 152 tumours occurred in non-smokers, 3 of which were parotid tumours and 2 paranasal sinus tumours, which are not strongly associated with smoking. Tobacco consumption was most strongly associated with the development of cancer of the larynx in this series of patients, but all the head and neck tumours with the exception of tumours of the nasopharynx and paranasal sinuses were significantly increased in male smokers.

Alcohol consumption was also much higher among male cases than controls. A significantly greater proportion of cases than controls reported drinking whiskey and wine in addition to beer, but this probably reflects the tendency of heavier drinkers, in particular "binge" drinkers, to use multiple beverages. Apart from these "mixed" drinkers, no differences were seen in the type of alcohol consumed. In his 1956 study of cancer of the larynx Wynder and his colleagues found that consumption of spirits was greater among cases than controls, but in a later study (1976) no significant differences in the type of alcohol consumed were found. Feldman & Hazan (1975) found no difference between cases and controls except for "mixed" drinkers, who predominated among the cases. It seems likely that it is the total quantity of alcohol consumed which is important in the development of cancers of the upper respiratory and alimentary tracts, but the possibility that "mixed" drinking increases the risk cannot be ruled out.

The question of whether alcohol *per se* is carcinogenic or whether it is a promoter of carcinogenesis remains unanswered. Wynder & Mabuchi (1973) point to the lack of experimental evidence that alcohol is carcinogenic, but recent studies (Breslow & Enstrom, 1974; Dean *et al.*, 1979) suggest that beer drinking may be a factor in the development of cancer of the rectum. In terms of prevention it seems that whatever its biochemical action, reduction in alcohol consumption would reduce the incidence of head and neck tumours. In this study the strongest association with alcohol consumption was seen in the development of cancer of the tongue, but increased risks were shown for all head and neck tumours in men.

Many studies (Wynder *et al.*, 1956; Feldman & Hazan, 1975; Jayant *et al.*, 1977; Simarak *et al.*, 1977; McMichael, 1978; Ward Hinds *et al.*, 1979) have shown that tobacco is an independent risk factor for neoplasms of the upper respiratory and upper gastrointestinal tracts, but the nature of the action of alcohol or of the combined effect of alcohol and tobacco is not so clear-cut. Since most heavy drinkers are also heavy smokers, the majority of investigators have not found a sizable group of cases who drank heavily but who were non-smokers or who smoked only lightly, and it has proved difficult, therefore, to separate precisely the effects of tobacco and alcohol consumption. Wynder *et al.* (1976) showed that at each measure of cigarette consumption heavy alcohol consumption increased the risk of laryngeal cancer, but no such increased risk was seen in non-smoking heavy drinkers. The findings of Feldman & Hazan (1975) were similar, but a significant synergistic effect between tobacco and alcohol was not demonstrated. In this series heavy drinking in the presence of non- and light smoking produced only a small increase in risk, but when combined with heavy smoking the risk rose sharply. However, statistically significant synergism was seen only in patients with cancer of the larynx.

The dental care of cases and controls was uniformly bad and the significantly higher number of denture wearers among the cases as compared to controls, was probably due to the fact that more cases than controls lived in urban areas and, therefore, had easier access to dental care.

The findings of this study confirm that cigarette smoking is the most important risk factor recognized for head and neck cancer, and suggested a synergistic action with heavy alcohol consumption. However, there can be no doubt that other aetiological factors must influence the development of some of these tumours; oropharyngeal cancer frequently occurs in the absence of exposure to tobacco or alcohol and is relatively more common in women than in men, and all the other tumours studied are known to occur in persons who never smoked or drank. The hypothesis that dietary deficiencies may influence the metabolism of epithelial cells is a plausible one, and deserves further study. However, the evidence that abstinence from smoking and moderation in alcohol consumption would produce substantial decreases in incidence and mortality from head and neck cancer is incontrovertible.

We are grateful to the Directors of St. Luke's Hospital, Dublin, for a grant for this study from the St. Luke's Cancer Research Fund, and to Professor M. J. O'Halloran, Dr F. H. Cross and Dr J. B. Healy, Consultant Oncologists, St. Luke's Hospital, for permission to interview patients under their care. We are also indebted to the staffs of the Radiotherapy and Medical Records' Department of St. Luke's Hospital and of the Computer Laboratory of University College, Dublin, for their courtesy and assistance.

### REFERENCES

BRESLOW, N. E. & ENSTROM, J. E. (1974) Geographic correlations between cancer mortality rates and alcohol–tobacco consumption in the United States. *J. Natl Cancer Inst.*, **53**, 631.

DEAN, G., MACLENNAN, R., MCLOUGHLIN, H. & SHELLEY, E. (1979) Causes of death of blue-collar workers at a Dublin brewery, 1954–73. *Br. J. Cancer*, **40**, 581.

FELDMAN, J. G. & HAZAN, M. (1975) A case-control investigation of alcohol, tobacco and diet in head and neck cancer. *Prev. Med.*, **4**, 444.

JAYANT, K., BALAKRISHNAN, V., SANGHVI, L. D. & JUSAWALLA, D. J. (1977) Quantification of the role of smoking and chewing tobacco in oral, pharyngeal and oesophageal cancer. *Br. J. Cancer*, **35**, 232.

MANTEL, N. & HAENSZEL, W. (1959) Statistical aspects of the analysis of data from retrospective studies of disease. *J. Natl Cancer Inst.*, **22**, 719.

MARTINEZ, I. (1970) Retrospective and prospective study of carcinoma of the oesophagus, mouth and pharynx in Puerto Rico. *Bull. Assoc. Med. Puerto Rico*, **62**, 170.

MCMICHAEL, A. J. (1978) Increases in laryngeal cancer in Britain and Australia, in relation to tobacco and alcohol consumption trends. *Lancet*, i, 1244.

ROTHAMN, K. J. (1974) Synergy and antagonism in cause effect relationships. *Am. J. Epidemiol.*, **99**, 385.

ROTHMAN, K. J. (1976) The estimation of synergy or antagonism. *Am. J. Epidemiol.*, **103**, 506.

ROTHMAN, K. & KELLER, A. Z. (1972) The effect of joint exposure to alcohol and tobacco on risk of cancer of the mouth and pharynx. *J. Chron. Dis.*, **26**, 711.

SIMARAK, S., DE JONG, V. W., BRESLOW, N. & 4 others (1977) Cancer of the oral cavity, pharynx, larynx and lung in North Thailand: Case-control study and analysis of cigar smoke. *Br. J. Cancer*, **36**, 130.

SURGEON GENERAL (1979) *Smoking and Health.* Washington: U.S. Department of Health, Education and Welfare.

VINCENT, R. G. & MARCHETTA, F. (1963) The relationship of the use of tobacco and alcohol to cancer of the oral cavity, pharynx or larynx. *Am. J. Surg.*, **106**, 501.

WARD HINDS, M., THOMAS, D. B. & O'REILLY, H. P. (1979) Asbestos dental x-rays, tobacco and alcohol in the epidemiology of laryngeal cancer. *Cancer*, **44**, 1114.

WYNDER, E. L., BROSS, I. J. & DAY, E. (1956) A study of environmental factors in cancer of the larynx. *Cancer*, **9**, 86.

WYNDER, E. L., BROSS, I. J. & FELDMAN, R. M. (1957) A study of the etiology factors in cancer of the mouth. *Cancer*, **10**, 1300.

WYNDER, E. L. & CHAN, P. C. (1970) The possible role of riboflavin deficiency in epithelial neoplasia. II. Effect on skin tumour development. *Cancer*, **26**, 1221.

WYNDER, E. L. & MABUCHI, K. (1973) Etiological and environmental factors in esophageal cancer. *J. Am. Med. Ass.*, **226**, 1546.

WYNDER, E. L., COVEY, L. S., MABUCHI, K. & MUSHINSKI, M. (1976) Environmental factors in cancer of the larynx: A second look. *Cancer*, **38**, 1591.